# Primary hepatic leiomyosarcoma concurrent with early-stage lung adenocarcinoma: a rare case report

**DOI:** 10.3389/fonc.2025.1621686

**Published:** 2025-07-01

**Authors:** Weizhong Peng, Xiaohui Xiao, Lu Li, Yi Yu

**Affiliations:** ^1^ Hepatobiliary Surgery, The First People’s Hospital of Chenzhou City, Chenzhou, Hunan, China; ^2^ Department of Ultrasound, Xiangnan University Affiliated Hospital, Chenzhou, Hunan, China; ^3^ Pathology Department, The First People’s Hospital of Chenzhou City, Chenzhou, Hunan, China

**Keywords:** primary hepatic leiomyosarcoma, lung adenocarcinoma, synchronous primary malignancies, liver neoplasms, multidisciplinary management

## Abstract

Primary hepatic leiomyosarcoma (PHL) is an extremely rare malignant mesenchymal tumor, accounting for less than 2% of all primary hepatic malignancies. We report a case of a 46-year-old female who presented with a one-year history of abdominal distension and pain. Imaging revealed a 6×5×4 cm mass in the left hepatic lobe (segments 3/4) and incidentally detected a suspicious pulmonary nodule. Laparoscopic partial left hepatectomy followed by thoracoscopic wedge resection of the left upper lung confirmed the diagnoses of primary hepatic leiomyosarcoma and primary lung adenocarcinoma (cT1aN0M0), respectively. This case highlights the importance of comprehensive diagnostics, immunohistochemical analysis, and multidisciplinary management of rare hepatic tumors, particularly when concurrent malignancies are present. This first reported case of concurrent PHL and primary lung adenocarcinoma provides valuable insights for clinical practice in managing patients with rare hepatic malignancies.

## Introduction

Primary hepatic leiomyosarcoma (PHL) is an exceedingly rare mesenchymal tumor originating from smooth muscle cells of the hepatic vasculature, bile ducts, or ligamentum teres (1). It constitutes less than 2% of all primary hepatic malignancies and approximately 0.5-2% of all primary liver sarcomas (2, 3). The etiology remains largely unclear, although associations with immunosuppression, Epstein-Barr virus infection, and previous chemotherapy exposure have been reported (4, 5).

The diagnosis of PHL presents significant challenges due to its rarity and nonspecific clinical and radiological features that often mimic more common hepatic neoplasms, particularly hepatocellular carcinoma (HCC) and intrahepatic cholangiocarcinoma (6, 7). Accurate diagnosis relies heavily on histopathological examination with comprehensive immunohistochemical analysis (8).

Synchronous primary malignancies in different organs occur with an estimated incidence of 0.7-11.7% (9, 10). However, the coincidence of primary hepatic leiomyosarcoma and primary lung adenocarcinoma represents an extremely rare occurrence in the literature.

This report presents a unique case of concurrent primary hepatic leiomyosarcoma and early-stage primary lung adenocarcinoma in a 46-year-old female. We describe our diagnostic approach, surgical management strategy, histopathological findings, and short-term outcomes, providing valuable insights for clinicians managing patients with rare hepatic malignancies.

## Case report

### Clinical presentation

A 46-year-old Chinese woman with no significant medical history (specifically no smoking, alcohol consumption, or hepatitis) presented to our hospital with a one-year history of progressive abdominal distension and pain, particularly exacerbated after meals. One year prior, abdominal computed tomography (CT) at a local hospital had revealed a hepatic mass considered benign, for which no specific intervention was recommended. The patient sought care at our institution due to worsening symptoms.

Physical examination revealed abdominal distension with deep tenderness in the upper abdomen and mild percussion pain in the hepatic region. Vital signs were stable: temperature 36.4°C, pulse 71 beats/minute, respiratory rate 20 breaths/minute, and blood pressure 126/81 mmHg. No jaundice, hepatosplenomegaly.

### Laboratory and imaging investigations

Laboratory investigations showed normal complete blood count, liver and renal function tests. Tumor markers, including carcinoembryonic antigen (0.37 ng/ml), alpha-fetoprotein (3.79 ng/ml), and protein induced by vitamin K absence-II (PIVKA-II, 25 mAu/ml), were within normal ranges.

**Figure 1:**
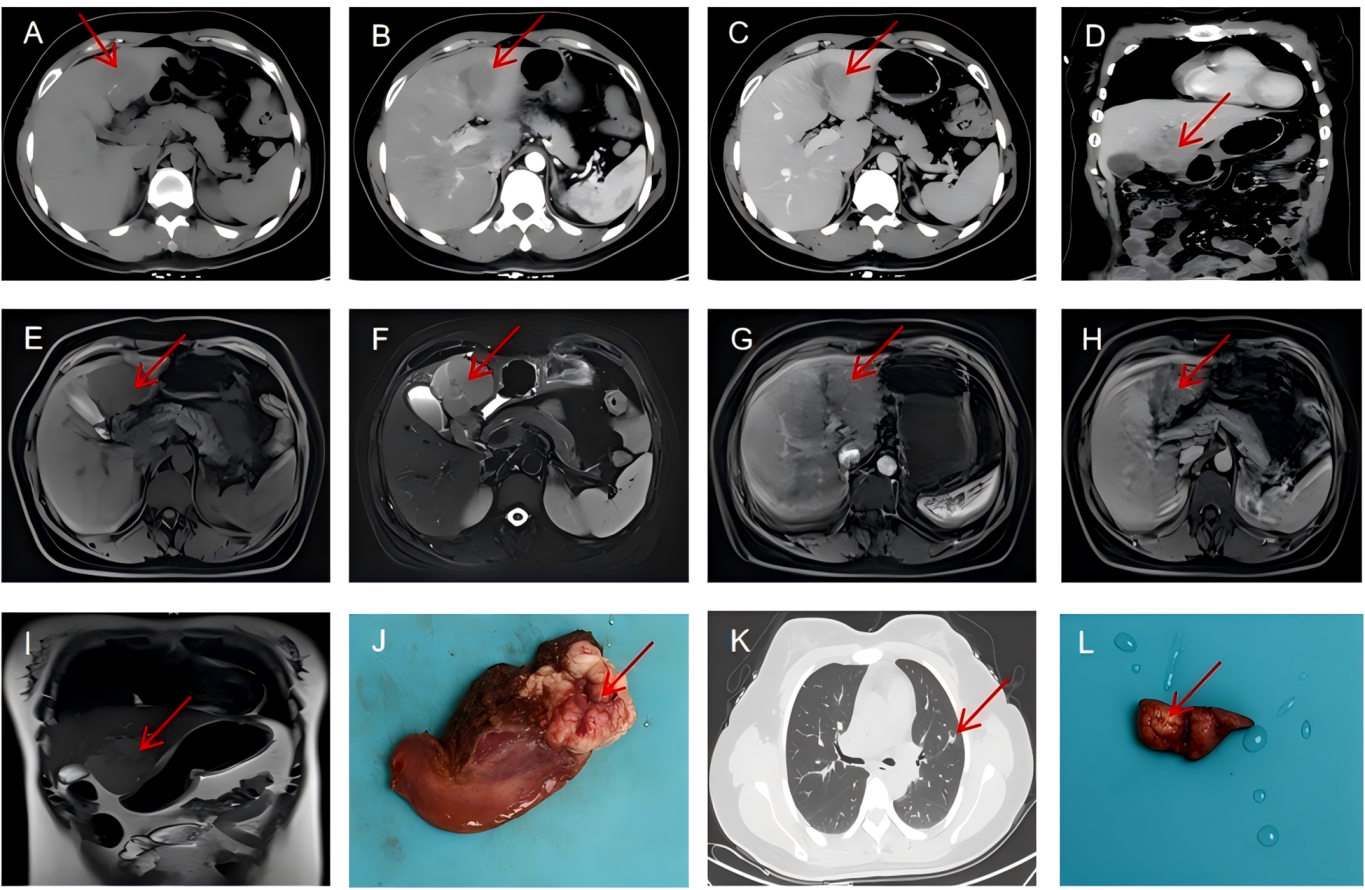
Radiological findings. **(A–D)** Contrast-enhanced CT scan showing a heterogeneous mass in liver segments 3 and 4 with peripheral enhancement (arrow). **(E)** The liver mass with hypointensity on T1-weighted images(arrow). **(F)** T2-weighted MRI demonstrating mild hyperintensity of the hepatic mass (arrow). **(G–I)** Hepatobiliary phase MRI showing hypointensity of the tumor with characteristic washout (arrow). **(J)** A gross specimen of the liver showed a round, brownish-white mass with foci of hemorrhage and necrosis. **(K)** Chest CT (lung window) showing the part-solid nodule in the left upper lobe with predominantly ground-glass appearance and small solid component (arrow). **(L)** Postoperative lung specimen showing a white mass of approximately 1.2 cm in diameter(arrow).

Enhanced CT scan of the abdomen revealed a well-demarcated, heterogeneously enhancing mass in liver segments 3 and 4, measuring approximately 6×5×4 cm with exophytic growth pattern (**Figures 1A–D**). The lesion demonstrated peripheral enhancement in the arterial phase with progressive central filling in the venous and delayed phases, initially raising suspicion for HCC or atypical hemangioma.

Chest CT incidentally identified a 14 mm part-solid nodule in the left upper lobe with a 2 mm solid component (CTR<0.5), classified as LU-RADS 4 (**Figure 1K**). Magnetic resonance imaging (MRI) with hepatocyte-specific contrast agent (gadoxetic acid) confirmed the liver mass with hypointensity on T1-weighted images (**Figure 1E**), mild hyperintensity on T2-weighted images(**Figure 1F**), and heterogeneous enhancement with characteristic washout in the hepatobiliary phase (**Figures 1G–I**). The lesion demonstrated restricted diffusion on diffusion-weighted imaging, further supporting malignancy. Gastroscopy and colonoscopy were unremarkable.

### Multidisciplinary team assessment

The case was presented at our multidisciplinary tumor board, comprising hepatobiliary surgeons, thoracic surgeons, oncologists, radiologists, and pathologists. After thorough discussion, the consensus was that both lesions likely represented separate primary malignancies rather than metastatic disease. Given the patient’s symptomatic hepatic mass and its larger size, the team recommended a staged approach with initial hepatic resection followed by pulmonary resection after adequate recovery.

### Surgical management and pathological findings

#### Hepatic resection

On December 25, 2024, the patient underwent laparoscopic partial left hepatectomy. The procedure was performed using a five-port technique. Intraoperative ultrasonography confirmed the tumor location in segments 3 and 4 without involvement of major vascular structures. A laparoscopic Cavitron Ultrasonic Surgical Aspirator (CUSA) was utilized to establish a 1-cm tumor-free margin. The parenchymal transection was performed with intermittent Pringle maneuver (15 minutes occlusion, 5 minutes reperfusion). The total operation time was 180 minutes with an estimated blood loss of 200 ml, requiring no transfusion.

**Figure 2:**
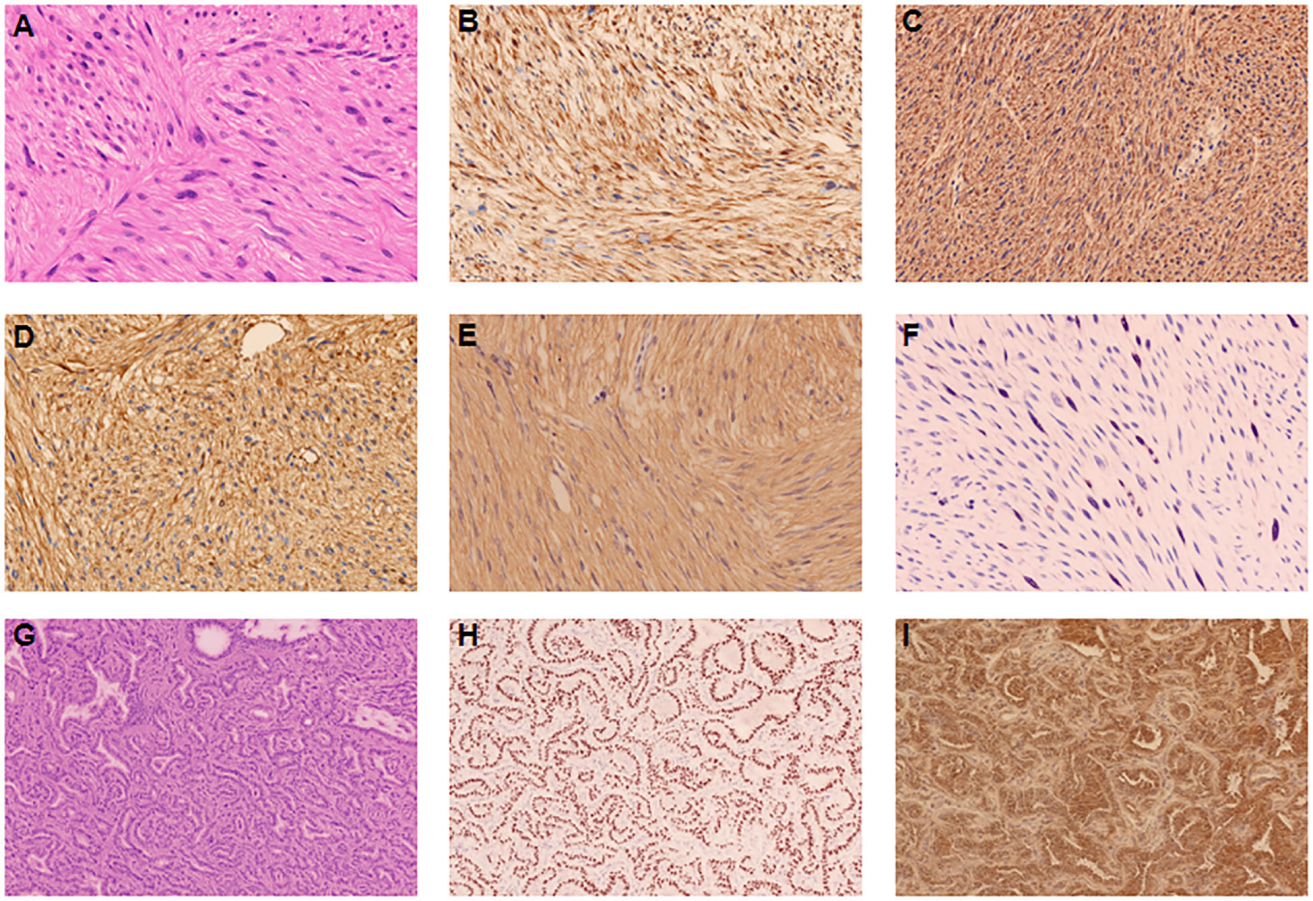
Histopathological and immunohistochemical findings of primary hepatic leiomyosarcoma **(A–F)**. **(A)** Hematoxylin and eosin staining showing intersecting fascicles of spindle cells with elongated nuclei and eosinophilic cytoplasm (magnification ×200). **(B–F)** Immunohistochemical staining showing strong positivity for SMA **(B)**, Desmin **(C)**, Vimentin **(D)**,Caldesmon **(E)**, and Ki-67 proliferation index of 20% **(F)**. Histopathological and immunohistochemical findings of primary lung adenocarcinoma **(G–I)**. **(G)** Hematoxylin and eosin staining showing adenocarcinoma with predominantly acinar pattern (magnification ×200). **(H, I)** Immunohistochemical staining showing strong positivity for TTF-1 **(H)** and Napsin A **(I)**.

Gross examination revealed a well-circumscribed, tan-white, firm mass measuring 6×5×4 cm with focal areas of hemorrhage and necrosis (**Figure 1J**). Histopathological examination showed intersecting fascicles of spindle cells with moderate nuclear pleomorphism, cigar-shaped nuclei, and eosinophilic cytoplasm (**Figure 2A**). Mitotic figures were identified (30 per 50 high-power fields), with focal areas of necrosis (approximately 10% of tumor volume).

Immunohistochemical analysis demonstrated strong positivity for smooth muscle actin (SMA) (**Figure 2B**), desmin (**Figure 2C**), vimentin (**Figure 2D**), and caldesmon (**Figure 2E**), with a Ki-67 proliferation index of 20% (**Figure 2F**). The tumor was negative for ALK, CK-pan, CD117, SATB2, STAT6, DOG-1, CD68, and estrogen receptor (ER). Only focal positivity for CD34 was observed. These findings established the diagnosis of primary hepatic leiomyosarcoma (histologic grade 2 according to the French Federation of Cancer Centers Sarcoma Group [FNCLCC] system).

The patient’s postoperative course was uneventful. She received standard postoperative care, including prophylactic antibiotics, thromboprophylaxis, and early mobilization. Liver function tests normalized by postoperative day 5, and she was discharged on January 7, 2025 (postoperative day 13). The patient was advised to undergo postoperative chemotherapy, which the patient refused to do. At the three-month follow-up, she remained asymptomatic with no evidence of disease recurrence on surveillance imaging.

#### Pulmonary resection

Following recovery from hepatic surgery, a follow-up PET/CT on February 17, 2025, confirmed postoperative changes in the liver without evidence of recurrence or metastasis but showed the persistent left upper lung nodule, consistent with primary lung adenocarcinoma.

The patient was admitted to the thoracic surgery department for further management. Chest CT confirmed a 12 mm part-solid nodule in the anterior segment of the left upper lobe with a solid component of 2 mm (CTR<0.5), clinically staged as cT1aN0M0 (**Figure 1K**).

On February 20, 2025, the patient underwent thoracoscopic wedge resection of the left upper lung with systematic hilar and mediastinal lymph node dissection using a three-port technique. Intraoperative frozen section analysis confirmed malignancy, and the resection was completed with adequate margins. The procedure lasted 110 minutes with minimal blood loss (approximately 50 ml).

Histopathological examination revealed a 1.2 cm invasive adenocarcinoma with predominantly acinar (70%) and lepidic (30%) patterns (**Figure 2G**), classified as grade 2 according to the International Association for the Study of Lung Cancer (IASLC) criteria. The tumor showed negative pleural invasion, vascular invasion, neural invasion, airway spread, and surgical margins. All lymph nodes (stations 5, 11, and 12) were negative for metastasis.

Immunohistochemistry demonstrated strong positivity for thyroid transcription factor-1 (TTF-1) (**Figure 2H**) and Napsin A (**Figure 2I**), while negative for ALK(1A4)(routinely performed to exclude ALK-rearranged lung adenocarcinoma, which would impact therapeutic considerations), CK5/6, and P63, confirming primary lung adenocarcinoma.

The patient recovered uneventfully and was discharged on February 24, 2025 (postoperative day 4).

## Discussion

### Primary hepatic leiomyosarcoma: diagnostic challenges and considerations

Primary hepatic leiomyosarcoma is one of the rarest primary liver malignancies, with fewer than 70 cases reported in the English literature (11). The challenge in diagnosis lies not only in its rarity but also in its nonspecific clinical and radiological features that often overlap with more common hepatic neoplasms (12).

In our case, the initial radiological impression suggested hepatocellular carcinoma, highlighting the limited specificity of imaging for this rare entity. Recent literature suggests that although certain radiological features may raise suspicion for PHL, including heterogeneous enhancement, central necrosis, and absence of typical HCC features (such as arterial hyperenhancement with portal/delayed phase washout), definitive diagnosis requires histopathological confirmation (13).

The importance of comprehensive immunohistochemical analysis cannot be overstated in the diagnosis of PHL. The tumor’s mesenchymal origin necessitates the demonstration of smooth muscle differentiation through positivity for SMA, desmin, and caldesmon, while excluding other sarcoma subtypes and metastatic disease (14). In our patient, the immunohistochemical profile was classic for leiomyosarcoma, with strong positivity for SMA, desmin, vimentin, and caldesmon, while negative for epithelial markers (cytokeratins), gastrointestinal stromal tumor markers (CD117, DOG-1), and other mesenchymal tumor markers (STAT6, SATB2).

Establishing the primary hepatic origin of leiomyosarcoma requires the exclusion of metastasis from other sites, particularly the gastrointestinal tract, retroperitoneum, and female genital tract. Our patient underwent comprehensive evaluations, including gastroscopy, colonoscopy, PET/CT, and gynecological examination, which excluded potential primary tumors elsewhere, supporting the diagnosis of PHL.

### Surgical management considerations for primary hepatic leiomyosarcoma

Complete surgical resection with negative margins remains the cornerstone of treatment for PHL, offering the best chance for long-term survival (15). The choice between open and laparoscopic approaches should be individualized based on tumor location, size, and relationship to vascular structures (16).

In our case, laparoscopic partial left hepatectomy was chosen due to the peripheral location of the tumor in segments 3 and 4, absence of major vascular involvement, and the surgeon’s expertise in minimally invasive liver surgery. The advantages of the laparoscopic approach include reduced postoperative pain, shorter hospital stay, and faster recovery, particularly relevant in our patient who required subsequent pulmonary surgery.

The surgical strategy for PHL should aim for R0 resection with a minimum margin of 1 cm when possible (17). While anatomical resection is generally preferred for HCC, non-anatomical parenchymal-sparing resection may be appropriate for PHL, particularly when located peripherally, as the tumor typically spreads by direct invasion rather than through portal venous dissemination (18). Our surgical approach aligned with these principles, achieving clear margins while preserving functional liver parenchyma.

The role of lymphadenectomy in PHL remains controversial due to limited data. Unlike HCC, where lymphatic spread is uncommon, sarcomas may metastasize via lymphatics, suggesting a potential benefit of regional lymphadenectomy (19). In our case, locoregional lymph nodes were sampled but showed no evidence of metastasis.

### Synchronous primary malignancies: management strategy and rationale

The occurrence of synchronous primary malignancies presents unique challenges in determining the optimal sequence and modalities of treatment. The decision-making process should consider the stage, potential aggressiveness, and symptomatology of each tumor, as well as the patient’s overall condition (20, 21).

In our case, the multidisciplinary team recommended addressing the hepatic tumor first, based on several considerations: (1) the hepatic tumor was larger and more symptomatic; (2) PHL generally has a more aggressive clinical course compared to early-stage lung adenocarcinoma; and (3) the pulmonary lesion was small with predominantly ground-glass appearance, suggesting indolent behavior.

This staged approach allowed adequate recovery between procedures and avoided the potential complications of simultaneous major surgeries. The successful outcome in our patient supports the value of this strategy, though it must be emphasized that treatment decisions for such rare dual malignancies should be individualized through multidisciplinary discussion.

### Pathogenetic considerations of double primary malignancies

The pathogenetic relationship between primary hepatic leiomyosarcoma and lung adenocarcinoma remains speculative due to the extreme rarity of this combination. Several theories might explain the occurrence of double primary malignancies:

1. Field cancerization: Exposure to common carcinogenic factors might affect multiple organs simultaneously, leading to independent primary tumors.
2. Genetic predisposition: Germline mutations in tumor suppressor genes or DNA repair genes might increase susceptibility to multiple malignancies.
3. Immune system dysfunction: Impaired immune surveillance might fail to eliminate malignant cells in multiple organs.
4. Random occurrence: Given the increasing incidence of cancer with age, the coincidence of two primary malignancies might represent a rare but statistically possible random event.

In our patient, the absence of known risk factors for either malignancy (no smoking history, hepatitis, or family history of cancer) suggests either unidentified environmental exposures, subtle genetic predisposition, or simply a rare coincidence. Molecular profiling of both tumors might provide insights into potential common pathogenetic mechanisms, representing an area for future research. Although molecular profiling using next-generation sequencing of both tumors could potentially provide valuable insights into common pathogenetic mechanisms, this was not performed in our case due to technical and financial limitations. Future studies incorporating comprehensive molecular characterization would be beneficial in understanding the genetic basis of such rare synchronous malignancies.

### Prognostic considerations and follow-up strategy

The prognosis of PHL is generally poor, with reported 5-year survival rates ranging from 19-37% (8). Recent registry analyses from the Surveillance, Epidemiology, and End Results (SEER) database suggest that complete surgical resection remains the most significant prognostic factor, with potentially improved outcomes in the era of advanced surgical techniques and targeted therapies (22).Factors associated with poor outcomes include tumor size >5 cm, high-grade histology, presence of necrosis, and positive surgical margins (23). In our patient, although the tumor was relatively large (6 cm) with focal necrosis, complete resection with negative margins was achieved, potentially improving her prognosis.

For early-stage lung adenocarcinoma (stage IA), the prognosis is generally favorable, with 5-year survival rates exceeding 80-90% following complete resection (24). The predominantly lepidic and acinar patterns observed in our patient’s tumor are associated with better outcomes compared to solid or micropapillary patterns.

The optimal surveillance strategy for patients with dual primary malignancies is not well established. We have implemented a tailored follow-up protocol for our patient, including:

1. First year: Clinical evaluation, liver function tests, and tumor markers every 3 months; chest and abdominal CT every 6 months.
2. Second to fifth years: Clinical evaluation, liver function tests, and tumor markers every 4–6 months; chest and abdominal CT every 6–12 months.
3. Beyond five years: Annual clinical evaluation with imaging as clinically indicated.

This intensive surveillance is designed to detect potential recurrence of either malignancy at an early, potentially treatable stage. The role of adjuvant therapy for either tumor in our patient remains uncertain given the complete resection and early-stage disease. Current evidence does not support routine adjuvant therapy for completely resected PHL (25) or stage IA lung adenocarcinoma (26). However, close monitoring for recurrence with consideration of systemic therapy at the first sign of disease progression represents a prudent approach.

### Limitations

Several limitations should be acknowledged in this case report. First, the relatively short follow-up period limits our ability to make definitive conclusions about long-term outcomes. Second, the patient’s refusal of adjuvant chemotherapy may impact disease-free survival, particularly for the hepatic leiomyosarcoma. Third, the lack of molecular profiling limits our understanding of potential genetic drivers and shared pathogenetic mechanisms between these two rare synchronous malignancies. Finally, as with all case reports, findings from a single patient may not be generalizable to the broader population of patients with similar presentations.

We acknowledge that immunohistochemical markers such as SMA, desmin, caldesmon, TTF-1, and Napsin A are not entirely specific to their respective tumor types. However, the divergent histological architecture, mutually exclusive marker expression, and discordant anatomical and clinical behavior of the two tumors argue strongly against a metastatic relationship. Although molecular profiling (e.g., NGS) could have provided additional confirmation, it was not performed due to financial and technical constraints. We recognize this as a limitation and propose that future studies of similar cases include genomic analysis to further delineate the origin of such rare synchronous tumors.

## Conclusion

This case report highlights the rare occurrence of primary hepatic leiomyosarcoma concurrent with primary lung adenocarcinoma, presenting several valuable clinical insights. First, it underscores the importance of comprehensive diagnostic evaluation, including detailed immunohistochemical analysis, in establishing the diagnosis of rare hepatic malignancies. Second, it demonstrates the crucial role of multidisciplinary collaboration in developing individualized treatment strategies for patients with synchronous primary malignancies. Third, it illustrates the feasibility and potential benefits of minimally invasive surgical approaches for both hepatic and pulmonary lesions in selected patients.

The successful sequential surgical management in our patient provides a potential roadmap for managing similar complex cases. Long-term follow-up is necessary to assess outcomes and refine management strategies for these exceedingly rare tumors. Future research directions should include molecular characterization of rare hepatic sarcomas and investigation of potential pathogenetic links between synchronous primary malignancies in different organs.

## Data Availability

The original contributions presented in the study are included in the article/supplementary material. Further inquiries can be directed to the corresponding authors.
